# Costs and constraints of cellular immune activity during development in an arboreal primate

**DOI:** 10.1098/rsos.241659

**Published:** 2025-04-02

**Authors:** Nicole Thompson González, Lucia Freedberg, James Higham, Erin Vogel, Marina Cords

**Affiliations:** ^1^ Integrative Anthropological Sciences, University of California Santa Barbara, Santa Barbara, CA, USA; ^2^ Department of Ecology, Evolution, and Environmental Biology, Columbia University, New York, NY, USA; ^3^ Department of Anthropology, New York University, New York, NY, USA; ^4^ Department of Anthropology, Rutgers University, New Brunswick, NJ, USA

**Keywords:** life history, ecoimmunology, conservation, immune ontogeny, energetics, energy balance

## Abstract

Evolutionary life history theory predicts that, during development, investment in immunity must be balanced with the demands of growth. How, and at what time scales, this balance is negotiated is unclear. In this study, we examined the potential energetic costs and limitations to cellular immune activity during development, its trade-offs with growth, related sickness behaviour and the role of hypothalamic-pituitary-adrenal (HPA) axis activity in these relationships. We combined biomarker and socio-environmental data on wild juvenile blue monkeys collected over eight months. Rather than detract from energy balance (C-peptide) and growth of lean body mass (creatinine by specific gravity residuals), cellular immune activity (neopterin) increased with energy balance and lean body mass at monthly time scales, suggesting an energetic constraint on cellular immunity. At shorter time scales, higher neopterin diminished subsequent growth. Energetic constraints on immune activity were weakly regulated by HPA activity during low energy states. Our results suggest that cellular immune activity is both costly and limited by physical condition in wild developing primates.

## 1. Introduction

Evolutionary life history theory predicts variation in the optimal relationships between energy allocation to growth, reproduction and maintenance throughout the life course [1]. In recent decades, the field of evolutionary and eco-immunology has demonstrated that immune function and activity also come at a significant energetic cost, placing immunity among the pillars of energetic investment within life history theory [2,3]. During development, physical growth is a high priority; however, it may trade off with the simultaneous necessity to defend against pathogens. Trade-offs between allocation to growth and immune function may be particularly intense when individuals are energetically constrained, such as in resource-limited environments, and during severe and/or prolonged infections. Successful growth has clear benefits; reduced adult body size can lower reproductive success [4] and compensatory growth can have significant implications for health [5,6] and survival [7], particularly in organisms with determinate growth [8]. Nevertheless, effective immune defences are essential for immediate survival in the face of pathogens. An area of obscurity, therefore, is the extent and time scales over which particular kinds of immunity (e.g. innate, cellular, humoral) bear costs and are prioritized relative to growth during development, and whether individual energetic condition constrains their activity. Lower energetic allocation to immune defence implies susceptibility to infection, therefore constraints on immunity and its potential trade-offs with growth have broad implications for individual and population health.

The costs of immune activity can be high. In terms of metabolic rates, experimental stimulation of adaptive immunity increased resting metabolic rate substantially in mice (20–30% [9]) and house sparrows (29% [10]). In humans, basal metabolic rate can increase by 7–14% with acute infections [11,12], while more severe infections can lead to increases up to 50% [12]. Costs also include behavioural changes, such as less physical activity, reduced feeding and increased sleep [13]. The additional costs of such sickness behaviour may include a loss of body mass, as in house sparrows with stimulated innate immunity [14]. Such compensatory sickness behaviour represents an opportunity cost of infection and is adaptively regulated by cytokine signals in an effort to heal and regain homeostasis [13].

Physical growth is a cornerstone of the juvenile period, as is the development of immunity to novel pathogens, and so competing investments in immunity and growth are likely to arise during this life stage. Mounting effective immune responses should be a high priority generally as survival in the face of pathogens is critical to lifetime fitness. However, the direction of trade-offs between immune activity and growth and the time scales at which they occur manifest differently according to the type, severity and duration of immune activity, coupled with individual energetic conditions. For example, among Shuar children in Amazonian Ecuador, acutely elevated c-reactive protein was associated with a 49% drop in growth over one week in thinner children, but at longer intervals (growth in lower leg length over 3 and 20 months, and height for age), and in children with more body fat there were no associated changes in growth [12]. Longer term investments in immunity, such as heightened immune surveillance or response to parasites, may probably compromise growth at longer intervals. Immune surveillance, as measured by non-acute concentrations of c-reactive protein (less than 2 mg l^−1^), corresponded with slower growth in adolescent Gambian women over one year (height velocity and lean mass deposition [15]). A broad literature also suggests that helminth parasites slow human growth, whereas antihelminthic treatment leads to gains in height and weight [16]. Similarly, experimental inoculation with parasites led to slower feather growth rates in juvenile barn swallows [17]. Finally, in reverse, a general prioritization of growth may result in weakened immune defence, as in domesticated chickens selected for faster growth that demonstrated weaker cellular and humoral immune responses [18].

Given its energetic costs, immune activity can also be energetically constrained, i.e. limited by available energy (intake and stores), nutrition and their associated signals. Several branches of the immune system depend on adequate caloric and nutrient intake and energy stores for proper function [19–21]. Fasting under experimental conditions suppresses T-cell-mediated immunity (humans [22]; Mongolian gerbils [23]), circulating concentrations of interferon-γ (IFN γ; rats [24]) and humoral immune function (Siberian hamsters [25]). Immunocompetence also varies in response to natural seasonal fluctuations in body fat stores in several seasonally breeding rodents [26]. Although energy balance and stores are generally immunomodulatory [27], the depletion of energy stores may compromise cellular immune function in particular. For example, fasted mice with lower body fat stores, indicated by lower circulating leptin concentrations, showed decreased cellular Th1 cytokines and a predominance of humoral Th2 cytokines, whereas upon refeeding they restored Th1 yet suppressed Th2 cytokines [28].

Although T-cell profiles require invasive sampling to quantify, cellular immune activity can be reliably measured non-invasively. The biomarker neopterin is a useful non-invasive marker of cellular immune activity, as well as general inflammatory activity of macrophages, as it is produced by type I macrophages that are primarily activated by Th1 cell-derived IFNy [29]. Neopterin has been shown to increase in response to acute infection in macaques [30] and great apes (bonobos [31]; chimpanzees [32]). It is excreted in urine and is highly stable when stored in the absence of light at low temperatures, making it an ideal marker of cellular immune activity in wild primate populations [33].

Energy balance and bodily condition are challenging to quantify in wild, large-bodied and arboreal mammals: the gold standard for measuring each requires capture or controls that are not feasible in most wild populations (e.g. doubly labelled water method). Nevertheless, the status of each can be approximated non-invasively. Energy balance, or the difference between energy intake and expenditure, can be well approximated with C-peptide of insulin [34–36]. C-peptide is excreted in urine and produced in a 1 : 1 ratio with insulin, providing an integrated signal of energy balance to the brain. C-peptide has been validated in several primate species, shown to correspond with within-individual variation in body mass (bonobos [37]; macaques [35]), feeding rates and mass gain and loss (macaques [35]) and food availability (chimpanzees [38]; orangutans [39]; gorillas [40]; blue monkeys [41]). Body condition typically refers to the availability of energetic reserves, namely body fat and lean muscle [42]. To date, no non-invasive methods have been validated to measure body fat; however, an approximation of lean body mass is possible with a combination of urinary creatinine and specific gravity [43]. Although lipids in adipose tissue are typically metabolized prior to protein in the lean muscle, the lean muscle still constitutes an energetic reserve [42,44]. Creatinine content of urine that exceeds expected values based on urine’s specific gravity indicates relatively higher muscle mass, and lower muscle mass if creatinine falls below expected values.

Several potential mechanisms mediate energetic constraints on the allocation of energy to immune function. Individuals may experience a direct lack of fuel for immune cells, such as glucose (rats [45]; mice [46]) or free fatty acids [47]. Such metabolic stress, however, can result in a general stress response, with the release of glucocorticoids (GCs) mediating the immunosuppressive effects of low energy. Indeed, concentrations of GCs rise in response to experimental fasting (Mongolian gerbils [23]) and in naturally occurring periods of low energy balance (blue monkeys [41]). Finally, depletion of body fat reduces circulating concentrations of leptin, a hormone similar in structure to IL-2 and similarly critical for sustaining cell-mediated immunity [2]. Though difficult to disentangle, understanding the mechanisms of energy-regulated immunomodulation is important for predicting circumstances in which immune function may be constrained and, more broadly, helps elucidate how life history trade-offs may be regulated (e.g. [48]).

Whether trade-offs between immune activity and growth occur in a given species and population probably depends on individual determinants of available energy and broader species’ life history. Higher dominance status in social animals often corresponds to the priority of access to resources, including calorically rich foods [49]. Therefore, individuals of higher status may evidence little trade-off as they have more energy to allocate towards both growth and immunity. Further, a fast rate of development and determinate growth may prioritize energetic allocations to growth, as short-term interruptions to growth could come at a higher cost to adult size or via catch-up growth [8]. In juvenile blue monkeys (*Cercopithecus mitis*), energy balance does not vary according to social status [41]. This species also demonstrates a particularly prolonged juvenile period even among primates, ranging from 1.5 to 10 years old, and a slow life history overall [50]. Given their suggested slow rate of growth and the weak or absent influence of dominance status on their energetics, we expected that juvenile blue monkeys would be able to withstand and recover from energetic shocks to growth, regardless of their status. Nevertheless, juvenile blue monkeys do experience energetic shortfalls, as their hypothalamic-pituitary-adrenal (HPA) axis activity is attuned to energy balance such that glucocorticoids increase during low energy states [41]. As such, slow growth may be an adaptive response to limited resource availability in their environment. We, therefore, expected to see limitations in allocations towards either cellular immunity or growth, depending on the extent of the costs of cellular immunity and the relative prioritization of growth.

Specifically, in this study, we examined the energetic costs and constraints associated with cellular immunity during juvenile development in wild blue monkeys. Our study had two main aims to study: (i) the energetic costs of cellular immune activity and (ii) the energetic constraints on cellular immune activity. If investment in cellular immune activity is costly and highly prioritized at the cost of growth, we expected that higher concentrations of urinary neopterin would correspond with lower C-peptide and smaller changes in lean body mass over time, and that trade-offs in body mass growth would be larger among individuals with lower versus higher energy balance. We further expected that the relationship between cellular immune activity and growth to vary at different time scales, such that short-term allocation to immunity, such as in response to acute infections, would compromise shorter (days to weeks) versus longer term (monthly) growth in lean muscle. Finally, if immune activity is energetically constrained, we expected that urinary neopterin would increase with energy balance and lean body mass. In the event of constraints, we aimed to examine whether immunity is limited by low energy balance itself or via the immunosuppressive effects of glucocorticoids.

## 2. Material and methods

### 2.1. Ethical statement

The Institutional Animal Care and Use Committee of Columbia University approved the data collection protocols for this study, which complied with the laws of Kenya. All research was conducted in accordance with these protocols and with permission of the Kenya Wildlife Service, National Environment Management Authority, National Commission for Science Technology and Innovation and Kenya Forest Service.

### 2.2. Study site and system

Blue monkeys are female philopatric arboreal guenons, endemic to Central and East Africa, that live in social groups with a single adult male, multiple adult females and their immature offspring. The study population resides in the Isecheno area of the Kakamega Forest, Kenya and is wild living and unprovisioned. Data were collected on 41 juveniles (20 females and 21 males, mean age 4.5 ± 1.7 yr) for eight months from August 2015 to March 2016. Subjects lived in three neighbouring social groups (mean group sizes: 37−65). Subjects were individually identifiable by their natural physical variation and their ages were known from precise, long-term demographic records on the study population [51]. A team of four observers including N.T.G. collected all biomarker and behavioural samples after a training period of two months to ensure reliable identification of all subjects.

### 2.3. Behavioural data collection

Observers in the field (including N.T.G.) conducted 20 min focal follows, during which they recorded a subject’s activity at 1 min intervals (e.g. resting, feeding, locomoting). The follows occurred between 07.30 and 17.00, and focal subjects were targeted for even progression in focal sampling over the month and during morning, midday and afternoon periods. In total, observers collected 1591 h of activity budget data, with an average of 39 ± 3.1 h per subject.

### 2.4. Non-invasive sample collection and assays

Biological samples were collected between the hours of 07.30 and 17.00 ad libitum after excretion by identified subjects. Additional sampling targeted subjects to ensure that each was sampled approximately once every two weeks, for a total of 614 urine (mean ± s.d., 2 ± 0.8 per subject/month) and 627 faecal samples (2 ± 0.7 per subject/month). Urinary neopterin (cellular immune activity), urinary C-peptide (uCP, energy balance) and faecal glucocorticoid metabolites (fGCs) were then measured using established protocols of enzyme immunoassay and within- and between-assay quality controls (see electronic supplementary material, section S1). Urinary creatinine was measured according to standard Jaffe reaction, and specific gravity (SG) was measured using a handheld refractometer.

### 2.5. Data analysis

Given circadian rhythms in physiological activity, we evaluated the effects of the time of sample collection on uCP, neopterin and fGC concentrations using a generalized linear mixed effects model (lme4 v. 1.1-31, lmerTest v. 3.1-3) with a gamma error distribution and log link. Marker concentrations were regressed against hours from midnight with random slopes and intercepts by subject ID. uCP was negatively correlated with time of day, consistent with other diurnal mammals [52], and so uCP concentrations were expressed as residuals from predicted values, derived from population average slopes and intercepts. Time of day was not included as a control within multivariate models because uCP, used primarily as a response variable, was expressed as an average monthly concentration to more broadly reflect individual energy balance. These time-adjusted residuals were made positive by adding to them the absolute value of the minimum time-adjusted residual and an infinitesimal value of 0.0001 to ensure values greater than 0 for log transformation.

**Figure 1.**
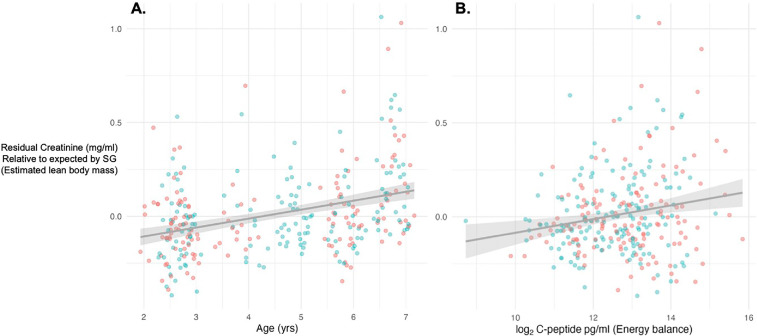
Validation relationships between (A) age and (B) monthly energy balance with monthly estimated lean body mass. Points represent raw values for individual monthly average creatinine residuals, relative to creatinine concentrations predicted by specific gravity in a linear regression. Blue points represent male juveniles, red points represent female juveniles.

We calculated estimated lean body mass (ELBM) as residuals of observed to expected creatinine concentrations for a given SG, according to the formula presented in Emery Thompson [43,53] and validated in multiple primate species (e.g. orangutans [44], chimpanzees [43]). Creatinine is produced at a steady rate within individuals and positively correlates with urinary SG to reflect urine dilution; however, deviations from expected creatinine concentrations for a given sample SG reflect the relative quantity of individual lean muscle mass. To estimate lean muscle mass, residuals of observed creatinine values were calculated relative to predicted creatinine values, derived from a linear regression of creatinine ng ml^−1^ predicted by linear and quadratic terms of SG (minus 1, [53]). Although creatinine is produced by energy metabolism in muscle, raw creatinine concentrations were unrelated to physical activities (i.e. ß ± s.e. time spent resting = −0.08 ± 0.27, feeding = 0.02 ± 0.55, locomoting = 0.08 ± 0.44, *p* > 0.05). Residual creatinine increased with age in both individual samples (ß age = 0.04 ± 0.008, *p* < 0.0001) and as monthly average residuals (ß age = 0.04 ± 0.009, *p* < 0.0001; figure 1A) and was unrelated to subject sex (sample: ß sex = 0.003 ± 0.03, *p* > 0.05: monthly average: ß sex = 0.004 ± 0.03 *p* > 0.05). Creatinine residuals also corresponded positively with uCP at the sample (ß uCP_sample_ = 0.013 ± 0.005, *p* < 0.05) and monthly average levels (ß uCP_month_ = 0.025 ± 0.01, *p* < 0.05; figure 1B). These results suggest that our calculation of ELBM represented an accurate estimate of lean body mass during development in this species.

To represent subjects’ general physiological state, urinary neopterin, uCP, fGCs and ELBM were averaged per subject by month for several analyses (tables 1 and 2). This time frame further allowed pairing between urinary markers, faecal markers and monthly activity budgets. To examine longer term effects of immune activity on growth, we calculated monthly change in lean body mass (∆ELBM_month_ = month_2_ – month_1_). To assess the short-term effects of immune activity on growth, we calculated the change in lean body mass by subtracting the ELBM of the given sample at *t*_1_ from the ELBM of the subsequent sample at *t*_2_ (∆ELBM_sample_ = *t*_2_ – *t*_1_, interval of varying lengths). Total averages and standard deviations in biomarker concentrations (neopterin, uCP, lean body mass and fGCs) are available in electronic supplementary material, table S1.

**Table 1.** Generalized linear mixed model structures examining aim 1: energetic costs of immune activity in *n* = 41 subjects.

costs model^a^	response	predictors	*n*
1. energy balance	log_2_ uCP_month_	age + sex + maternal rank + log_2_ neopterin_month_	293 subject months
2. monthly growth	∆ELBM_month_	age + sex + maternal rank + log_2_ neopterin_month_ + log_2_ uCP_month_	252 subject monthly ∆*s*
2a. energy-dependent monthly growth	∆ELBM_month_	age + sex + maternal rank + log_2_ neopterin_month_ + quartiles uCP_month_ + log_2_ neopterin_month_ : quartiles uCP_month_	252 subject monthly ∆*s*
3. shorter term growth	∆ELBM_sample *t*2 – *t*1_	age + sex + maternal rank + log_2_ neopterin_t1_ + log_2_ uCP_t1_ + interval_t2-t1_ + log2 neopterin_t1_ : interval_t2-t1_ + log2 uCP_t1_ : interval_t2-t1_	520 subject sample ∆*s*
4−6. compensatory behaviour	monthly time resting, feeding or locomoting	age + sex + maternal rank + log_2_ neopterin_month +_ log2 uCP_month_	293 subject months

^a^ All models include subject ID and month as random effects.

**Table 2.** Generalized linear mixed model structures examining aim 2: energetic constraints on immune activity in *n* = 41 subjects.

constraints model^a^	response	predictors	*n*
7. constraints on immune activity^b^	log_2_ neopterin_month_	age + sex + maternal rank + log_2_ uCP_month_ + ELBM_month_ + rainfall_month_ + N social partners_month_	293 subject months
*fGC mediation of constraints:*	—	—	288 subject months
8a. total effect	log_2_ neopterin_month_	age + sex + log_2_ uCP_month_	—
8b. effect on mediator	log_2_ fGCs_month_	age + sex + log_2_ uCP_month_	—
8c. direct effect	log_2_ neopterin_month_	age + sex + log_2_ uCP_month_ + fGC_month_	—

^a^ All models include subject ID as a random effect.

^b^ Also includes month as a random effect.

Juvenile dominance rank is socially inherited from mothers in this species [54] and female rank is highly stable from year to year [55]. We therefore calculated individual maternal dominance rank using the I&SI method in DomiCalc by MC [56] based on clear winner–loser interactions using data collected during the study period if mothers were still alive, or during the mother’s last year of life. Maternal rank was expressed as the proportion of group females that a mother outranked, ranging from 0 to 1. Subject age was calculated at the mid-date of the month. Subjects’ times spent resting, feeding and locomoting were calculated as the proportion of 1 min point samples of total observation time per month that subjects were engaged in that behaviour.

Finally, to statistically isolate the influence of physical condition (i.e. energy balance and estimated lean body mass) on energetic allocations to cellular immune activity, we aimed to control for seasonal and social variables that are likely to contribute to immune marker variation resulting from pathogen exposure. Rainfall is known to be a major driver of seasonal parasitic infection (57) and the number of social partners is a driver of respiratory infectious disease risk [58]. Monthly rainfall was calculated as a sum of local daily rainfall records in millimetres, collected by the Kenya Forest Service. Individuals’ total number of social partners was extracted from focal data and included unique partners with whom individuals rested in proximity, sat in contact, played and groomed.

### 2.6. Statistical analysis

1. *Energetic costs of immune activity*. To evaluate the potential energetic costs of cellular immune activity, we constructed three linear mixed effects regression models using the lme4 (v. 1.1-31) and lmerTest (v. 3.1-3) packages (table 1). All analyses were run in R v. 4.2.1. Models 1–2a evaluated costs at broad levels of energy balance and growth. These included subjects’ monthly average concentrations of uCP and monthly growth (month_2_ − month_1_ average ELBM) as responses, with subject age, sex, maternal rank and monthly average neopterin concentrations as fixed effects. Model 2a further included the quartile of an individual’s monthly average uCP as a main effect and in interaction with neopterin, to evaluate whether trade-offs between cellular immune activity and growth were stronger for individuals with the lowest versus highest energy balance. All models included random effects for subject ID and month to capture additional seasonal influences on biomarkers. We then examined the short-term effects of immune activity on growth, using ΔELBM_sample_ as an outcome and sample neopterin concentration at *t*_1_, age and maternal rank as fixed effects (model 3). These models included the time interval between samples at *t*_1_ and *t*_2_ (mean ± s.d., 14 ± 11 days) as an independent fixed effect and in interaction with neopterin, as we expected the influence of acute concentrations in neopterin to wane with greater time between sampling. To further evaluate if juvenile behaviour responded to the energetic demands of immune activity in a compensatory way, we evaluated monthly time spent resting, feeding and locomoting as responses to average neopterin, age, sex and maternal rank (models 4–6).
2. *Energetic constraints on immune activity*. We constructed a model where energy balance and lean body mass predicted neopterin concentrations (model 7). Here, we further controlled for total monthly rainfall (mm) and a subject’s number of social partners in the same month, as potential proxies of pathogen exposure [59–61]. Given the strong positive effects of energy balance on neopterin in this model, we further explored whether low energy balance may restrict neopterin production via its inverse relationship with anti-inflammatory glucocorticoids. We constructed mediation models that included sub-models of the total effect of energy balance on neopterin (sub-model 8a), the effect of energy balance on the potential mediator fGCs (sub-model 8b) and the direct effect of energy balance on neopterin, controlling for fGCs (sub-model 8c). We used the mediation package in R, which takes sub-models 8b and 8c as arguments, to evaluate the proportion of the total effect of energy balance on neopterin that is accounted for by the relationship between energy balance with fGCs and fGCs’ subsequent relationship with neopterin.

In all models, average and raw sample concentrations of uCP and neopterin were scaled by log base 2, to bring them onto similar scales of other predictors and to easily interpret their effect on the response. This means that a doubling of a predictor value increases the response by ß. For parsimony, we also maintained variable values on a log_2_ scale as responses to normalize their distributions. Where models including all individuals indicated significant costs or constraints on immune activity, we further examined the estimated coefficients of key predictor variables within individuals to evaluate the consistency of the directionality of their effects.

## 3. Results

### 3.1. Energetic costs of immune activity

**Figure 2.**
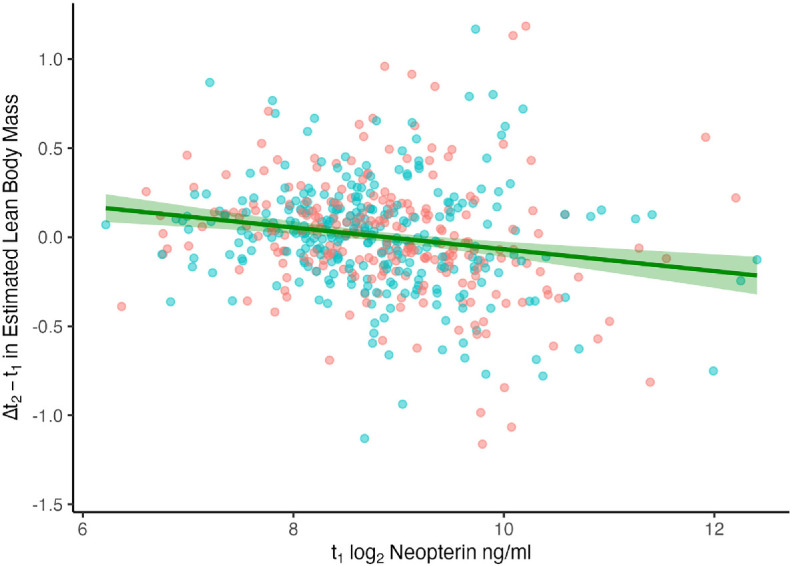
Decrease of short-term growth (estimated lean body mass at sample *t*_2_*− t*_1_) with cellular immune activity (neopterin concentrations at sample *t*_1_) using LMM, *n* = 520. Points indicate subject-sample values for males (blue) and females (red).

**Figure 3.**
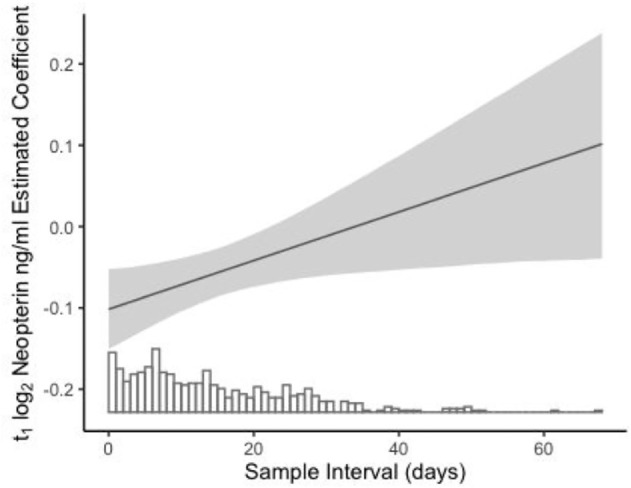
Change in the model estimated coefficient (LMM) of sample *t*_1_ neopterin concentrations on the subsequent short-term growth (sample *t*_2_ − *t*_1_ ELBM) as the interval of *t1*_1_ − *t*_2_ increases. Bar rug represents the frequency of intervals of a given length. The negative effect of neopterin on the subsequent growth fades as the sample interval becomes longer.

Counter to predictions of the energetic costs and prioritization of cellular immune activity, we found that neopterin concentrations had a positive relationship with monthly energy balance (uCP, table 3) and found no evidence that higher monthly concentrations of neopterin predicted lower monthly growth (table 3), even among individuals with low energy balance (electronic supplementary material, table S2). Nevertheless, at shorter time scales, higher neopterin concentrations at sample *t*_1_ resulted in smaller subsequent changes in ELBM at *t*_2_ (table 3, figure 2). Consistent with an acute effect of immune activity on growth, the relationship between sample neopterin and short-term growth faded as the interval between sampling times became longer (figure 3). The relationship between neopterin and short-term growth, alongside an absence of neopterin’s influence on monthly growth, suggests compensation in growth after acute rises in inflammation subside. The negative relationship between short-term changes in body mass and neopterin concentrations was present in most within-individual analyses, 26 of 41, where the mean slope and its mean standard error were −0.15 ± 0.27. Finally, in line with sickness behaviour, higher concentrations of monthly neopterin corresponded with increased time spent resting (ß = 0.014 ± 0.005, *p* = 0.003, electronic supplementary material, table S3). Neopterin concentration had no relationship with monthly time spent feeding or locomoting.

**Table 3.** Costs of immune activity: results from linear mixed models 1, 2 and 3. Significant relationships in bold.

response	predictor	estimate	s.e.	CI	*p*-value
avg. C-peptide *n* = 293	intercept	9.532	0.708	[8.14,10.92]	<0.001
	sex	−0.302	0.155	[−0.61,0]	0.059
	age	0.088	0.047	[0,0.18]	0.07
	maternal rank	−0.062	0.251	[−0.55,0.43]	0.806
	**log_2_ avg neopterin**	**0.328**	0.075	[0.18,0.48]	**<0.001**
∆ ELBM_month2 - 1_ *n* = 252	intercept	0.617	0.237	[0.15,1.08]	0.01
	age	0.008	0.009	[−0.01,0.03]	0.372
	sex	−0.023	0.031	[−0.08,0.04]	0.444
	maternal rank	0.009	0.049	[−0.09,0.1]	0.846
	log2 avg. neopterin	−0.011	0.021	[−0.05,0.03]	0.587
	**log_2_ avg. C-peptide**	**−0.040**	0.015	[−0.07,−0.01]	**0.01**
∆ ELBM_sample *t*2 *− t*1_ *n* = 520	intercept	1.027	0.247	[0.54,1.51]	<0.001
	age	0.011	0.008	[0,0.03]	0.195
	sex	−0.005	0.027	[−0.06,0.05]	0.862
	maternal rank	0.011	0.043	[−0.07,0.1]	0.802
	**log_2_ neopterin**_*t*1_	**−0.102**	0.025	[−0.15,−0.05]	**<0.001**
	log_2_ C-peptide_*t*1_	−0.019	0.016	[−0.05,0.01]	0.248
	**sample interval**_*t*2 − *t*1_	**−0.033**	0.013	[−0.06,−0.01]	**0.012**
	**log_2_ neo**_*t*1_ **× sample interval**	**0.003**	0.001	[0,0.01]	**0.023**
	log_2_ CP_*t*1_ × sample interval	0.001	0.001	[0,0]	0.27

### 3.2. Energetic constraints on immune activity

**Figure 4.**
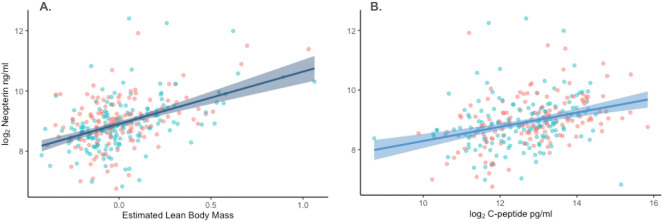
(A) Increase in cellular immune activity (neopterin ng ml^−1^) with estimated lean body mass (monthly average creatinine-SG residual). (B) Increase in cellular immune activity with energy balance (monthly average C-peptide ng ml^−1^). Results using LMM, *n* = 293. Points indicate subject-month values for males (blue) and females (red).

**Figure 5.**
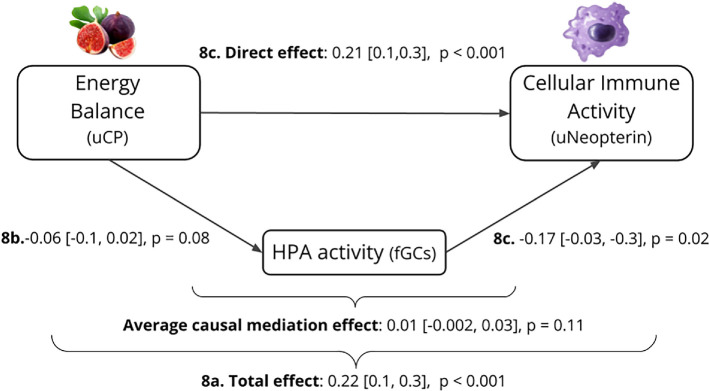
Mediation analysis of fGCs’ role in the relationship between energy balance and cellular immune activity. Beta coefficients and (95% confidence intervals) for models 8a–c and average causal mediation effect from R function ‘mediate’.

We found that both energy balance and estimated lean body mass had strong positive relationships with neopterin, independent of subject age, sex, maternal rank and potential pathogen exposure (figure 4A,B, table 4). None of these variables demonstrated a clear relationship with average neopterin concentrations. The positive relationship between neopterin and energy balance, and neopterin and lean body mass, was present in most within-individual analyses, 30 and 33 of 41 respectively, where the mean slopes and their mean standard errors were 0.16 ± 0.33 and 1.8 ± 1.8. Mediation analysis revealed that most of the total effect of uCP on neopterin was accounted for by their direct relationship. Only 4% (95% CI 0.1–14%) of variation in average neopterin concentrations was accounted for by glucocorticoids, suggesting a weak mediating role of the anti-inflammatory effects of GC secretion during states of low energy balance (figure 5; electronic supplementary material, table S4).

**Table 4.** Constraints and drivers of cellular immune activity: results of LMM, *n* = 293 subject months. Significant relationships in bold.

response	predictor	estimate	s.e.	CI	*p*-value
avg. neopterin	intercept	7.144	0.641	[5.89,8.4]	<0.001
	sex	−0.041	0.099	[−0.23,0.15]	0.678
	age	0.001	0.032	[−0.06,0.06]	0.975
	maternal rank	0.051	0.158	[−0.26,0.36]	0.748
	**ELBM**	**1.447**	0.239	[0.98,1.91]	**<0.001**
	**log_2_ C-peptide**	**0.14**	0.042	[0.06,0.22]	**0.001**
	monthly rainfall	−0.016	0.031	[−0.08,0.05]	0.630
	N social partners	0.007	0.01	[−0.01,0.03]	0.476

## 4. Discussion

In this study, we aimed to evaluate the energetics of cellular immune activity during development in a wild primate, considering both its potential energetic costs and constraints. Evidence of the costs of cellular immunity was limited to short-term growth: elevated concentrations of neopterin were unrelated to growth from month to month; however, they corresponded with less growth over shorter time intervals. Juveniles with higher neopterin rested more but did not change time spent feeding or locomoting. In addition to its cost in relation to short-term growth, cellular immune activity appeared to be limited by individual physical condition: both estimated lean body mass (creatinine-SG residual) and energy balance (urinary C-peptide) corresponded positively with neopterin concentrations, independently of any individual attribute, or environmental and social factors. This apparent energetic constraint on cellular immunity was only weakly mediated by anti-inflammatory effects of glucocorticoids that increased in low-energy conditions. Overall, these energetic dynamics of cellular immune activity suggest that cellular immunity is not generally costly to growth during development in blue monkeys and can in fact be energetically constrained.

Cellular immune activity, as measured by concentrations of urinary neopterin, did not influence changes in estimated lean body mass from month to month in juvenile blue monkeys, regardless of individual energetic condition; however, it did acutely inhibit growth. Increased resting did not account for lower short-term growth, as creatinine excretion, which served as the basis of lean body mass estimates, was unrelated to physical activity. The time scale of neopterin’s influence on growth in lean body mass suggests that developmental growth was able to compensate quickly for short-term setbacks. Despite a general gap in animal physiological literature on the time scale of immune costs to developmental growth, these results do align with literature demonstrating that growth is a high priority during development and not particularly encumbered by inflammatory immune activity. In chickens selected for enhanced immune function, there was little observed trade-off with growth [18]. In Shuar forager-horticulturalists, inflammatory innate immune activity also showed trade-offs in childhood height velocity over one week intervals; however, it did not affect longer term changes in height [12]. Nevertheless, humoral immune activity in response to parasitic infection did affect Shuar children’s growth at longer time scales (growth over 3 and 20 months and height for age [12]) and, among Tsimane forager-horticulturalists, greater energetic allocations to cellular immune activity as measured by Th1 cell counts corresponded with lower height for age [62]. This contrary evidence highlights how different measures of immunity may represent differential degrees and durations of energetic allocations to immune function, as both humoral immune activity and cell counts may evidence larger investments in the development and maintenance of immune defences, relative to more acute energetic allocations evidenced by macrophage activity (neopterin).

While costly, cellular immune activity appeared constrained by the physical condition in juvenile blue monkeys. Lower energy balance and lean body mass both corresponded with lower neopterin levels. This relationship was independent of seasonal and social variables that represent potential pathogen exposure, i.e. monthly rainfall and an individual’s number of social partners, which were unrelated to neopterin concentrations. Although seasonal effects on immune function are abundant in the animal literature, evidence for a direct effect of energetic constraints is rare. For example, prairie voles decrease body mass and IgG production during short-day photoperiods [63]; however, these effects are probably driven by seasonal reproductive regression and corresponding changes in androgens [63,64]. Other animals, such as Siberian hamsters, have demonstrated a direct effect of energy on immunity, such that experimental reductions in circulating energy availability directly reduced humoral immune function [25]. Similarly, in healthy and non-obese human adults, caloric restriction lowers total white blood cell and lymphocyte counts [65].

An alternative explanation for the positive relationship observed between immune activity and energy balance could involve the consumption of foods that are energetically dense yet found in areas that pose high infection risk, such as anthropogenic food sources [66]. Although better nutrition from calorically dense foods may augment healthy immune function, initial exposures to anthropogenic pathogens nevertheless heighten immune activity. Heightened cellular immune activity after exposures could be particularly likely for young individuals that are encountering many pathogens for the first time. In this study’s population, the home ranges of multiple study groups overlap with a forest station and tree nursery that contains a large oil palm with high-fat fruits [67]. When feeding in this area, animals may consume abundant calories; however, they are exposed to anthropogenic disturbance and potential one-on-one interactions with humans. Future analyses could analyse whether the relationship between immune activity and energy balance is indirectly driven, in part, by the differential use of habitats and the nutritional content of diets consumed therein.

The relationship between energy balance and cellular immune activity in juvenile blue monkeys was not substantially mediated by the immunosuppressive effects of elevated glucocorticoids during low energy states, similar to results found for Siberian hamsters [25]. This evidence is consistent with the hypothesis that low energy, in the form of low glucose, may directly constrain immune activity; however, other mediating factors could be at play. For example, fasting leads to lower levels of circulating leptin in rodents [23,28], which is essential for the maintenance of cell-mediated immunity [2]. The mediating role of adipose tissue and leptin has yet to be evaluated in the study population.

It is possible that energetic limitations on cellular immune activity are a particular risk for developing individuals, given the high priority given to physical growth during development. During adulthood, a primary competing demand for immunity and somatic maintenance is reproduction, which can be delayed or terminated [68] with arguably fewer impacts on lifetime fitness than stunted growth [4,69] or the costs associated with compensatory growth [8,70]. Furthermore, juveniles may be particularly susceptible to the negative consequences resulting from energetic constraints on immune activity because of their immunological naivety [59] and their corresponding need to sustain higher white blood cell counts than adults [71]. For this reason, a population or social group with a greater number of juveniles may overall become more susceptible to disease spread during food scarcity [59].

## 5. Limitations of the study

Our study is limited by the difficulty of differentiating immune activity and immune function *per se* in an observational study, by the limited growth time frame of eight months, and further by the scope of immune activity examined, i.e. cellular. Juveniles’ true infection status or pathogen exposure was unknown, therefore low concentrations of markers for immune activity could indicate a relatively healthy state, free of infection or an immunosuppressed state, which would respond insufficiently to infection. Independent markers of infection status (e.g. experimentally induced infection, antigen-specific antibodies) and immune function (e.g. challenge tests), would allow for a more precise evaluation of the costs of activity and energetic limitations on function. Nevertheless, two aspects of this study bolster our interpretations. First, monthly average markers of cellular immune activity provide a more integrative measure of investment in immune activity than single samples. Second, we control for environmental and social dimensions of pathogen exposure in our analysis of the energetic constraints of cellular immune activity (tables 2 and 4). Together, these methods and controls allow us a degree of confidence that individuals in poorer physical condition, i.e. with lower body mass for a given age and energy balance, face energetic limitations to cellular immune activity and function. Nevertheless, energetic allocation, costs and constraints of innate and humoral immune activity may differ from these patterns and do so at different time windows. Developmental growth occurs over years, which was not captured in the eight month window of this study. Accordingly, we did not measure truly long-term consequences of growth either in relation to cellular or other relevant types of immune activity. Furthermore, a lack of evidence of individual susceptibility to infection also hinders a nuanced analysis of the role of exposures in immune activity [72].

The mechanisms of energetic constraints on cellular immunity during development in blue monkeys are still unclear. Our findings suggest that glucocorticoids are not a primary mediator responsible for lower neopterin with lower energy balance; however, we do not rule out their role entirely. Measures of urinary glucocorticoids, rather than faecal metabolites, would allow for finer temporal pairings between markers of HPA axis activity and urinary markers of energy balance and immune activity, to potentially reveal stronger associations and immunosuppression. Further future research to develop non-invasive techniques of measuring adipose tissue and circulating leptin will aid in determining whether depleted fat stores are critical to limiting cellular immunity in wild mammal populations.

## 6. Conclusions

We find here that while cellular immune activity appears to pose weak costs to developmental growth over days and weeks for an arboreal primate species, it does not bear great costs to developing individuals at monthly time scales. Furthermore, cellular immunity appears to be constrained by aspects of physical condition, including energy balance and estimated lean body mass. Individual physical condition played a stronger role in levels of immune activity than other individual, ecological or socio-environmental factors. Our study raises implications for the conservation of wild populations in seasonal and resource poor environments, particularly those with a high proportion of immature individuals. The costs and constraints of immune activity and its relationship to physical condition during development, broadly, will probably depend on the life history of a species, such that species with relatively prolonged developmental periods may be more resistant to energetic limitations on achieving healthy adult size, however, they may nevertheless be susceptible to infection in low versus high energy states. The mechanisms of energetic constraints on immunity during development in this study are uncertain. Fine-scale temporal pairing of measures of HPA activity with energy balance and lean body mass will help determine the role of glucocorticoids in immunosuppression. Further research to develop non-invasive techniques of measuring adipose tissue and circulating leptin will aid in determining whether depleted fat stores are critical to limiting cellular immunity in wild mammal populations. Examining these relationships between energy balance, growth and immune activity will be valuable to identify potential threats to individual and population level health.

## Data Availability

All relevant data are available at [73]. Analytical scripts are available at [74,75]. Supplementary material is available online [76].
